# The State Board of Health

**Published:** 1903-10

**Authors:** 


### THE STATE BOARD OF HEALTH.

THE recently appointed members of the State Board of Health met in Atlanta and organized for work. Dr. Willis F. Westmoreland, of Atlanta, was elected president; Dr. Chas. Hicks, of Dublin, vice-president, and Dr. H. F. Harris, of Atlanta, secretary. The last named office carries with it a salary of $2,000 a year. The secretary is practically the State health officer, acting under the authority of the board. Dr. Harris's qualifications and ability and well known attainments insure for the board an officer of rare efficiency, especially when assisted by such men as constitute the board as counsellors.

---

THE Tri-State Medical Society of Tennessee, Alabama and Georgia meets in Atlanta, October 13, 14, 15. Dr. Michael Hoke is president, and Dr. Willis Westmoreland, chairman of the local committee of entertainment. An attractive program has been arranged, and there is every prospect of a large and interesting meeting. Atlanta is glad to welcome the visiting members.

